# 1′-Butyl-2-methyl-1′,2,2′,3,4,9-hexa­hydro­spiro­[benzo[*f*]isoindole-1,3′-indole]-2′,4,9-trione

**DOI:** 10.1107/S1600536812026748

**Published:** 2012-06-20

**Authors:** G. Jagadeesan, K. Sethusankar, G. Bhaskar, P. T. Perumal

**Affiliations:** a Department of Physics, Dr MGR Educational and Research Institute, Dr MGR University, Chennai 600 095, India; b Department of Physics, RKM Vivekananda College (Autonomous), Chennai 600 004, India; c Organic Chemistry Division, Central Leather Research Institute, Adyar, Chennai 600 020, India

## Abstract

In the title compound, C_24_H_22_N_2_O_3_, the indoline and pyrrole-fused naphtho­quinone units are both essentially planar [r.m.s. deviations = 0.042 (3) and 0.133 (3) Å, respectively]. The pyrrole ring adopts a C-envelope conformation. The dihedral angle between the mean planes of the two five-membered rings is 89.94 (9)°. The O atoms deviate from the mean planes of the pyrrolidine and naphthalene rings by 0.0311 (2), 0.2570 (2) and 0.1669 (2) Å. In the crystal, C—H⋯O inter­actions generate dimers with *R*_2_^2^(16) and *R*_2_^2^(18) graph-set motifs. The carbonyl O atom is involved in bifurcated hydrogen bonding. C—H⋯π inter­actions also occur.

## Related literature

For the biological activity of indole derivatives, see: Stevenson *et al.* (2000 ▶); Rajeswaran *et al.* (1999 ▶); Amal Raj *et al.* (2003 ▶). For a related structure, see: McSweeney *et al.* (2004) ▶. For graph-set notation, see: Bernstein *et al.* (1995 ▶).
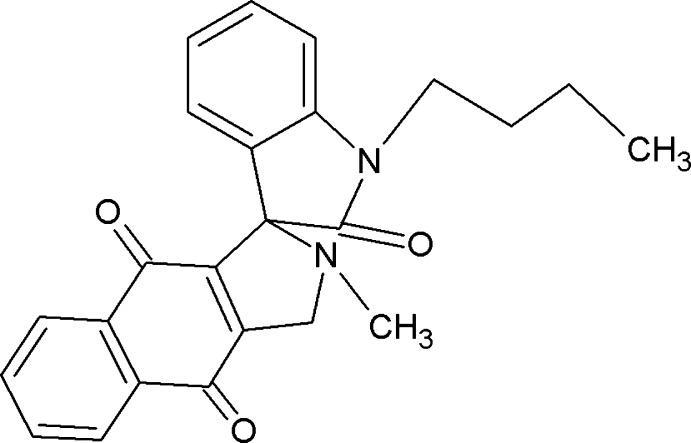


## Experimental

### 

#### Crystal data

C_24_H_22_N_2_O_3_
*M* *_r_* = 386.44
Monoclinic, 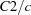

*a* = 21.5855 (12) Å
*b* = 15.7999 (7) Å
*c* = 14.7469 (7) Å
β = 127.207 (3)°
*V* = 4005.7 (3) Å^3^
*Z* = 8
Mo *K*α radiation
μ = 0.09 mm^−1^
*T* = 293 K
0.30 × 0.30 × 0.25 mm

#### Data collection

Bruker APEXII CCD area-detector diffractometer
Absorption correction: multi-scan (*SADABS*; Bruker, 2008 ▶) *T*_min_ = 0.975, *T*_max_ = 0.979
32353 measured reflections
3336 independent reflections
2446 reflections with *I* > 2σ(*I*)
*R*_int_ = 0.031

#### Refinement

*R*[*F* ^2^ > 2σ(*F* ^2^)] = 0.052
*wR*(*F* ^2^) = 0.178
*S* = 1.00
3336 reflections
264 parameters
H-atom parameters constrained
Δρ_max_ = 0.53 e Å^−3^
Δρ_min_ = −0.26 e Å^−3^

### 

Data collection: *APEX2* (Bruker, 2008 ▶); cell refinement: *SAINT* (Bruker, 2008 ▶); data reduction: *SAINT*; program(s) used to solve structure: *SHELXS97* (Sheldrick, 2008 ▶); program(s) used to refine structure: *SHELXL97* (Sheldrick, 2008 ▶); molecular graphics: *ORTEP-3* (Farrugia, 1997 ▶); software used to prepare material for publication: *SHELXL97* and *PLATON* (Spek, 2009 ▶).

## Supplementary Material

Crystal structure: contains datablock(s) global, I. DOI: 10.1107/S1600536812026748/pv2556sup1.cif

Structure factors: contains datablock(s) I. DOI: 10.1107/S1600536812026748/pv2556Isup2.hkl

Supplementary material file. DOI: 10.1107/S1600536812026748/pv2556Isup3.cml

Additional supplementary materials:  crystallographic information; 3D view; checkCIF report

## Figures and Tables

**Table 1:** Hydrogen-bond geometry (Å, °) *Cg*1 is the centroid of the C1–C6 ring.

*D*—H⋯*A*	*D*—H	H⋯*A*	*D*⋯*A*	*D*—H⋯*A*
C1—H1⋯O1^i^	0.93	2.39	3.232 (4)	151
C9—H9*A*⋯O2^ii^	0.97	2.45	3.230 (3)	137
C20—H20⋯O1^iii^	0.93	2.52	3.205 (3)	131
C11—H11*B*⋯*Cg*1^iv^	0.97	2.82	3.759 (4)	164

Symmetry codes: (i) 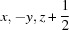; (ii) 
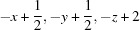
; (iii) 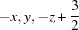; (iv) 
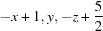
.

**Fig. 1.**
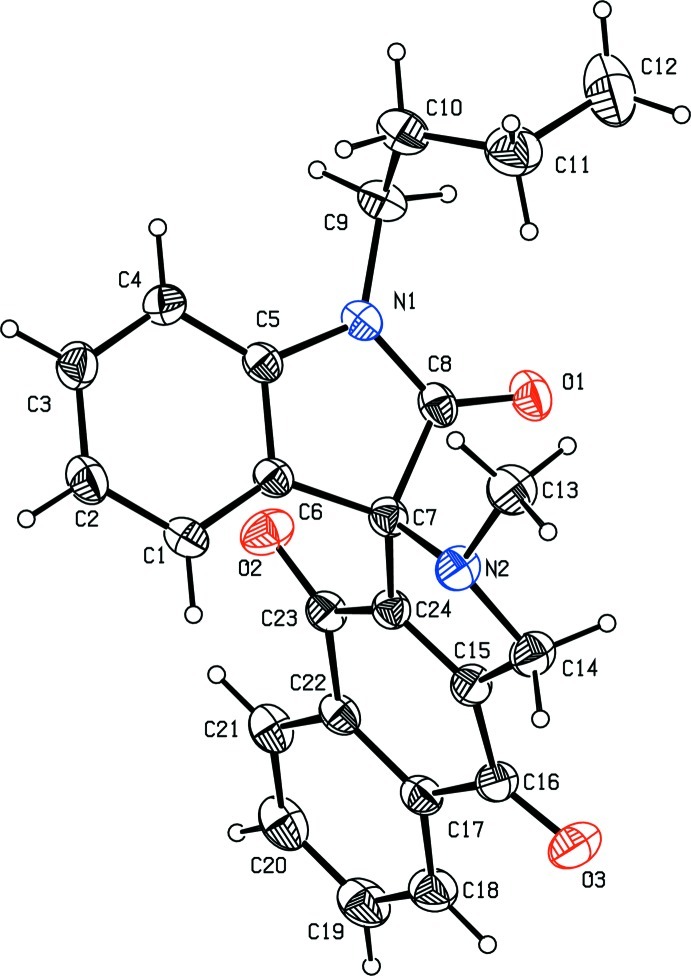
The molecular structure of the title compound showing 30% probability displacement ellipsoids.

**Fig. 2.**
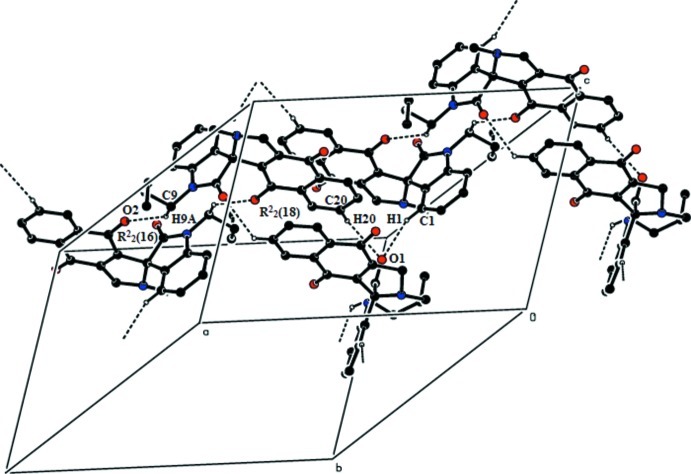
A view of the intermolecular hydrogen bonds (dotted lines) in the crystal structure of the title compound. H atoms non participating in H-bonding were ommitted for clarity.

## supplementary crystallographic information

### Comment

Indole compounds can be used as bioactive drugs (Stevenson *et al.*, 2000) and are also proven to display high aldose reductase inhibitory activity (Rajeswaran *et al.*, 1999), antimicrobial and antifungal activities (Amal Raj *et al.*, 2003).

In the title molecule (Fig. 1), the indoline moiety (N1/C1–C8) is essentially planar with rmsd 0.042 (3) Å. The pyrrole fused naphthoquinone moiety (N2/C7/C14–C24) is also planar with rmsd 0.133 (3) Å. The dihedral angle between the two five membered rings (C5/C6/C7/C8/N1) and (C7/C14/C15/C24/N2) is 89.94 (9) °.

The molecular structure of the title compound C_24_H_22_N_2_O_3_, is shown in Fig. 1. The dihedral angle between the two five membered rings (C5/C6/C7/C8/N1) and (C7/C14/C15/C24/N2) is 89.94 (9) °. The five-membered ring (C7/C14/C15/C24/N2) adopts a C13-envelope conformation with C13 0.142 (3) Å out of the plane formed by the rest of the ring atoms. The atom O1 deviates from the mean plane of the pyrrolidine ring (C5/C6/C7/C8/N1) by 0.0311 (2) Å. The atoms O2 and O3 deviate from the mean plane of the naphthalene ring (C15–C24) by 0.2570 (2) Å and 0.1669 (2) Å, respectively. The title compound exhibits the structural similarities with the already reported related structure (McSweeney *et al.*, 2004).

The crystal packing is stabilized by intermolecular C—H···O and C—H···π interactions. The C9—H9A···O2 and C20—H20···O1 hydrogen bonds generate dimers *R*^2^_2_(16) and *R*^2^_2_(18) graph set motifs, respectively (Bernstein, *et al.*, 1995); the carbonyl-group O1 atom is involved in bifurcated hydrogen bonding (Tab. 1 & Fig. 2). The crystal packing is further stabilized by C11—H11B···*Cg*1 interaction where *Cg*1 is center of gravity of (C1–C6) ring.

### Experimental

A mixture of napthaquinone (1 mmol), 1-butylisatin (1.05 mmol), and sarcosine (1.1 mmol), were stirred at 353 K about for 90 minutes. The reaction mixture was poured into water and extracted with ethyl acetate (2x25 ml). The combined extract was dried over anhydrous Na_2_SO_4_ and concentrated in vacuum. The resulting product was purified by column chromatography on silica gel (Merck, 60–120 mesh, ethyl acetate-hexane, 3:7) to afford the pure product which was subjected to crystallization by slow evaporation of a solution of ethanol resulting in single crystals of the title compound suitable for XRD studies.

### Refinement

The H atoms were placed at calculated positions in the riding model approximation with C—H = 0.93, 0.96 and 0.97 Å for aryl, methyl and methylene H-atoms, respectively, with *U*_iso_(H) = 1.5*U*_eq_(methyl C) and 1.2*U*_eq_(non-methyl C). The rotation angles for methyl groups were optimized by least squares.

### Crystal data

**Table:**

C_24_H_22_N_2_O_3_	*F*(000) = 1632
*M**_r_* = 386.44	*D*_x_ = 1.282 Mg m^−^^3^
Monoclinic, *C*2/*c*	Mo *K*α radiation, λ = 0.71073 Å
Hall symbol: -C 2yc	Cell parameters from 3336 reflections
*a* = 21.5855 (12) Å	θ = 2.6–24.5°
*b* = 15.7999 (7) Å	µ = 0.09 mm^−^^1^
*c* = 14.7469 (7) Å	*T* = 293 K
β = 127.207 (3)°	Block, colourless
*V* = 4005.7 (3) Å^3^	0.30 × 0.30 × 0.25 mm
*Z* = 8	

### Data collection

**Table:**

Bruker APEXII CCD area-detector diffractometer	3336 independent reflections
Radiation source: fine-focus sealed tube	2446 reflections with *I* > 2σ(*I*)
Graphite monochromator	*R*_int_ = 0.031
ω scans	θ_max_ = 24.5°, θ_min_ = 2.6°
Absorption correction: multi-scan (*SADABS*; Bruker, 2008)	*h* = −24→25
*T*_min_ = 0.975, *T*_max_ = 0.979	*k* = −18→18
32353 measured reflections	*l* = −17→17

### Refinement

**Table:**

Refinement on *F*^2^	Primary atom site location: structure-invariant direct methods
Least-squares matrix: full	Secondary atom site location: difference Fourier map
*R*[*F*^2^ > 2σ(*F*^2^)] = 0.052	Hydrogen site location: inferred from neighbouring sites
*wR*(*F*^2^) = 0.178	H-atom parameters constrained
*S* = 1.00	*w* = 1/[σ^2^(*F*_o_^2^) + (0.1024*P*)^2^ + 2.8296*P*] where *P* = (*F*_o_^2^ + 2*F*_c_^2^)/3
3336 reflections	(Δ/σ)_max_ < 0.001
264 parameters	Δρ_max_ = 0.53 e Å^−^^3^
0 restraints	Δρ_min_ = −0.26 e Å^−^^3^

### Special details

**Table:**

Geometry. All e.s.d.'s (except the e.s.d. in the dihedral angle between two l.s. planes) are estimated using the full covariance matrix. The cell e.s.d.'s are taken into account individually in the estimation of e.s.d.'s in distances, angles and torsion angles; correlations between e.s.d.'s in cell parameters are only used when they are defined by crystal symmetry. An approximate (isotropic) treatment of cell e.s.d.'s is used for estimating e.s.d.'s involving l.s. planes.
Refinement. Refinement of *F*^2^ against ALL reflections. The weighted *R*-factor *wR* and goodness of fit *S* are based on *F*^2^, conventional *R*-factors *R* are based on *F*, with *F* set to zero for negative *F*^2^. The threshold expression of *F*^2^ > σ(*F*^2^) is used only for calculating *R*-factors(gt) *etc*. and is not relevant to the choice of reflections for refinement. *R*-factors based on *F*^2^ are statistically about twice as large as those based on *F*, and *R*- factors based on ALL data will be even larger.

### Fractional atomic coordinates and isotropic or equivalent isotropic displacement parameters (Å^2^)

**Table:**

	*x*	*y*	*z*	*U*_iso_*/*U*_eq_	
C1	0.29896 (13)	0.09126 (16)	1.2560 (2)	0.0614 (6)	
H1	0.2836	0.0457	1.2781	0.074*	
C2	0.32375 (15)	0.16635 (19)	1.3171 (2)	0.0729 (7)	
H2	0.3246	0.1715	1.3807	0.088*	
C3	0.34705 (16)	0.23335 (18)	1.2847 (2)	0.0726 (7)	
H3	0.3634	0.2831	1.3269	0.087*	
C4	0.34673 (14)	0.22839 (15)	1.1912 (2)	0.0627 (6)	
H4	0.3627	0.2737	1.1697	0.075*	
C5	0.32181 (12)	0.15374 (14)	1.13096 (18)	0.0528 (5)	
C6	0.29768 (12)	0.08615 (14)	1.16220 (18)	0.0517 (5)	
C7	0.27195 (12)	0.01504 (14)	1.07856 (18)	0.0530 (5)	
C8	0.28365 (13)	0.05586 (15)	0.99368 (19)	0.0570 (6)	
C9	0.34151 (14)	0.18855 (18)	0.9826 (2)	0.0682 (7)	
H9A	0.3220	0.2454	0.9752	0.082*	
H9B	0.3202	0.1682	0.9068	0.082*	
C10	0.42958 (16)	0.1916 (2)	1.0533 (3)	0.0835 (8)	
H10A	0.4439	0.2389	1.0275	0.100*	
H10B	0.4509	0.2023	1.1320	0.100*	
C11	0.4655 (2)	0.1146 (2)	1.0486 (3)	0.1005 (10)	
H11A	0.4417	0.0655	1.0556	0.121*	
H11B	0.5204	0.1142	1.1123	0.121*	
C12	0.4562 (3)	0.1083 (3)	0.9369 (4)	0.1450 (17)	
H12A	0.4026	0.1172	0.8736	0.217*	
H12B	0.4722	0.0532	0.9311	0.217*	
H12C	0.4878	0.1506	0.9363	0.217*	
C13	0.39189 (15)	−0.06776 (18)	1.1579 (3)	0.0791 (8)	
H13A	0.3876	−0.0662	1.0893	0.119*	
H13B	0.4171	−0.1193	1.1984	0.119*	
H13C	0.4219	−0.0202	1.2049	0.119*	
C14	0.26445 (14)	−0.13602 (16)	1.0647 (2)	0.0708 (7)	
H14A	0.2738	−0.1827	1.1144	0.085*	
H14B	0.2716	−0.1555	1.0092	0.085*	
C15	0.18559 (13)	−0.09920 (15)	1.0084 (2)	0.0585 (6)	
C16	0.11206 (14)	−0.14631 (16)	0.94742 (19)	0.0608 (6)	
C17	0.04098 (13)	−0.09477 (16)	0.90004 (18)	0.0575 (6)	
C18	−0.03032 (16)	−0.13453 (19)	0.8497 (2)	0.0751 (7)	
H18	−0.0335	−0.1933	0.8458	0.090*	
C19	−0.09628 (16)	−0.0870 (2)	0.8056 (2)	0.0864 (9)	
H19	−0.1438	−0.1138	0.7722	0.104*	
C20	−0.09242 (15)	−0.0007 (2)	0.8106 (2)	0.0816 (8)	
H20	−0.1373	0.0308	0.7807	0.098*	
C21	−0.02266 (14)	0.03991 (18)	0.8594 (2)	0.0679 (7)	
H21	−0.0205	0.0987	0.8621	0.081*	
C22	0.04459 (12)	−0.00674 (15)	0.90473 (17)	0.0544 (6)	
C23	0.11946 (13)	0.03774 (15)	0.95638 (18)	0.0557 (6)	
C24	0.18925 (12)	−0.01533 (14)	1.01543 (18)	0.0517 (5)	
N1	0.31470 (11)	0.13398 (12)	1.03225 (15)	0.0557 (5)	
N2	0.31480 (11)	−0.06413 (12)	1.12956 (18)	0.0637 (5)	
O1	0.26952 (10)	0.02136 (12)	0.90967 (14)	0.0752 (5)	
O2	0.12328 (10)	0.11375 (12)	0.94839 (17)	0.0800 (6)	
O3	0.11025 (11)	−0.22243 (12)	0.93678 (18)	0.0870 (6)	

### Atomic displacement parameters (Å^2^)

**Table:**

	*U*^11^	*U*^22^	*U*^33^	*U*^12^	*U*^13^	*U*^23^
C1	0.0563 (13)	0.0760 (16)	0.0590 (13)	−0.0028 (11)	0.0386 (11)	0.0025 (12)
C2	0.0712 (16)	0.094 (2)	0.0621 (14)	−0.0040 (14)	0.0448 (13)	−0.0125 (14)
C3	0.0728 (16)	0.0749 (17)	0.0730 (16)	−0.0092 (13)	0.0455 (14)	−0.0187 (13)
C4	0.0618 (14)	0.0595 (14)	0.0696 (15)	−0.0063 (11)	0.0413 (12)	−0.0041 (12)
C5	0.0454 (11)	0.0626 (14)	0.0508 (12)	0.0019 (9)	0.0292 (10)	0.0007 (10)
C6	0.0413 (11)	0.0606 (13)	0.0526 (12)	0.0003 (9)	0.0281 (10)	−0.0001 (10)
C7	0.0468 (11)	0.0573 (13)	0.0564 (12)	0.0012 (9)	0.0320 (10)	−0.0013 (10)
C8	0.0451 (12)	0.0731 (16)	0.0541 (13)	0.0041 (10)	0.0306 (10)	−0.0044 (11)
C9	0.0639 (15)	0.0821 (17)	0.0672 (14)	0.0016 (12)	0.0441 (13)	0.0125 (13)
C10	0.0699 (17)	0.101 (2)	0.0929 (19)	−0.0033 (15)	0.0563 (16)	0.0024 (16)
C11	0.084 (2)	0.107 (3)	0.115 (3)	−0.0042 (18)	0.063 (2)	0.003 (2)
C12	0.150 (4)	0.175 (4)	0.137 (3)	0.007 (3)	0.101 (3)	−0.034 (3)
C13	0.0517 (14)	0.0852 (19)	0.0931 (19)	0.0096 (12)	0.0399 (14)	0.0035 (15)
C14	0.0615 (15)	0.0609 (15)	0.0879 (18)	0.0039 (11)	0.0441 (14)	0.0010 (13)
C15	0.0546 (13)	0.0578 (14)	0.0625 (13)	−0.0003 (10)	0.0351 (11)	−0.0022 (11)
C16	0.0642 (15)	0.0571 (15)	0.0627 (14)	−0.0050 (11)	0.0392 (12)	−0.0010 (11)
C17	0.0549 (13)	0.0713 (15)	0.0502 (12)	−0.0077 (11)	0.0339 (11)	−0.0039 (11)
C18	0.0649 (16)	0.0880 (19)	0.0735 (16)	−0.0184 (14)	0.0423 (14)	−0.0115 (14)
C19	0.0568 (16)	0.124 (3)	0.0774 (18)	−0.0200 (16)	0.0404 (15)	−0.0171 (18)
C20	0.0521 (15)	0.121 (3)	0.0659 (16)	0.0077 (15)	0.0326 (13)	−0.0033 (16)
C21	0.0536 (14)	0.0880 (18)	0.0578 (14)	0.0083 (12)	0.0315 (12)	0.0010 (12)
C22	0.0477 (12)	0.0713 (15)	0.0448 (11)	0.0013 (10)	0.0283 (10)	0.0014 (10)
C23	0.0550 (13)	0.0609 (15)	0.0529 (12)	0.0029 (10)	0.0335 (11)	0.0030 (10)
C24	0.0484 (12)	0.0550 (13)	0.0524 (12)	0.0002 (9)	0.0308 (10)	−0.0016 (10)
N1	0.0547 (11)	0.0645 (12)	0.0546 (10)	−0.0010 (9)	0.0365 (9)	0.0006 (9)
N2	0.0489 (11)	0.0605 (12)	0.0736 (13)	0.0066 (8)	0.0328 (10)	0.0030 (10)
O1	0.0700 (11)	0.0974 (13)	0.0652 (10)	−0.0079 (9)	0.0446 (9)	−0.0194 (10)
O2	0.0657 (11)	0.0588 (11)	0.1013 (14)	0.0054 (8)	0.0430 (10)	0.0115 (9)
O3	0.0802 (13)	0.0600 (12)	0.1113 (15)	−0.0097 (9)	0.0528 (12)	−0.0038 (10)

### Geometric parameters (Å, º)

**Table:**

C1—C6	1.369 (3)	C12—H12B	0.9600
C1—C2	1.386 (4)	C12—H12C	0.9600
C1—H1	0.9300	C13—N2	1.451 (3)
C2—C3	1.376 (4)	C13—H13A	0.9600
C2—H2	0.9300	C13—H13B	0.9600
C3—C4	1.376 (3)	C13—H13C	0.9600
C3—H3	0.9300	C14—N2	1.459 (3)
C4—C5	1.375 (3)	C14—C15	1.489 (3)
C4—H4	0.9300	C14—H14A	0.9700
C5—C6	1.384 (3)	C14—H14B	0.9700
C5—N1	1.403 (3)	C15—C24	1.328 (3)
C6—C7	1.505 (3)	C15—C16	1.468 (3)
C7—N2	1.464 (3)	C16—O3	1.211 (3)
C7—C24	1.508 (3)	C16—C17	1.486 (3)
C7—C8	1.558 (3)	C17—C22	1.392 (3)
C8—O1	1.212 (3)	C17—C18	1.390 (3)
C8—N1	1.355 (3)	C18—C19	1.377 (4)
C9—N1	1.459 (3)	C18—H18	0.9300
C9—C10	1.519 (4)	C19—C20	1.365 (4)
C9—H9A	0.9700	C19—H19	0.9300
C9—H9B	0.9700	C20—C21	1.373 (4)
C10—C11	1.467 (5)	C20—H20	0.9300
C10—H10A	0.9700	C21—C22	1.387 (3)
C10—H10B	0.9700	C21—H21	0.9300
C11—C12	1.536 (5)	C22—C23	1.482 (3)
C11—H11A	0.9700	C23—O2	1.214 (3)
C11—H11B	0.9700	C23—C24	1.464 (3)
C12—H12A	0.9600		
			
C6—C1—C2	118.1 (2)	H12B—C12—H12C	109.5
C6—C1—H1	121.0	N2—C13—H13A	109.5
C2—C1—H1	121.0	N2—C13—H13B	109.5
C3—C2—C1	120.7 (2)	H13A—C13—H13B	109.5
C3—C2—H2	119.6	N2—C13—H13C	109.5
C1—C2—H2	119.6	H13A—C13—H13C	109.5
C2—C3—C4	121.5 (2)	H13B—C13—H13C	109.5
C2—C3—H3	119.2	N2—C14—C15	102.05 (19)
C4—C3—H3	119.2	N2—C14—H14A	111.4
C3—C4—C5	117.3 (2)	C15—C14—H14A	111.4
C3—C4—H4	121.3	N2—C14—H14B	111.4
C5—C4—H4	121.3	C15—C14—H14B	111.4
C4—C5—C6	121.7 (2)	H14A—C14—H14B	109.2
C4—C5—N1	128.0 (2)	C24—C15—C16	123.0 (2)
C6—C5—N1	110.27 (19)	C24—C15—C14	110.6 (2)
C1—C6—C5	120.6 (2)	C16—C15—C14	126.3 (2)
C1—C6—C7	130.1 (2)	O3—C16—C15	121.4 (2)
C5—C6—C7	109.27 (19)	O3—C16—C17	122.7 (2)
N2—C7—C6	114.30 (18)	C15—C16—C17	115.9 (2)
N2—C7—C24	100.92 (17)	C22—C17—C18	119.2 (2)
C6—C7—C24	117.14 (18)	C22—C17—C16	120.9 (2)
N2—C7—C8	114.02 (18)	C18—C17—C16	119.9 (2)
C6—C7—C8	100.99 (18)	C19—C18—C17	120.0 (3)
C24—C7—C8	110.01 (17)	C19—C18—H18	120.0
O1—C8—N1	126.4 (2)	C17—C18—H18	120.0
O1—C8—C7	124.9 (2)	C20—C19—C18	120.5 (3)
N1—C8—C7	108.61 (18)	C20—C19—H19	119.7
N1—C9—C10	112.7 (2)	C18—C19—H19	119.7
N1—C9—H9A	109.1	C19—C20—C21	120.4 (3)
C10—C9—H9A	109.1	C19—C20—H20	119.8
N1—C9—H9B	109.1	C21—C20—H20	119.8
C10—C9—H9B	109.1	C20—C21—C22	120.0 (3)
H9A—C9—H9B	107.8	C20—C21—H21	120.0
C11—C10—C9	114.7 (3)	C22—C21—H21	120.0
C11—C10—H10A	108.6	C21—C22—C17	119.7 (2)
C9—C10—H10A	108.6	C21—C22—C23	119.6 (2)
C11—C10—H10B	108.6	C17—C22—C23	120.67 (19)
C9—C10—H10B	108.6	O2—C23—C24	121.2 (2)
H10A—C10—H10B	107.6	O2—C23—C22	122.4 (2)
C10—C11—C12	111.9 (3)	C24—C23—C22	116.4 (2)
C10—C11—H11A	109.2	C15—C24—C23	122.3 (2)
C12—C11—H11A	109.2	C15—C24—C7	110.99 (19)
C10—C11—H11B	109.2	C23—C24—C7	126.4 (2)
C12—C11—H11B	109.2	C8—N1—C5	110.76 (18)
H11A—C11—H11B	107.9	C8—N1—C9	124.99 (19)
C11—C12—H12A	109.5	C5—N1—C9	124.2 (2)
C11—C12—H12B	109.5	C13—N2—C14	115.3 (2)
H12A—C12—H12B	109.5	C13—N2—C7	116.1 (2)
C11—C12—H12C	109.5	C14—N2—C7	109.86 (18)
H12A—C12—H12C	109.5		
			
C6—C1—C2—C3	−0.6 (4)	C20—C21—C22—C23	179.5 (2)
C1—C2—C3—C4	−0.1 (4)	C18—C17—C22—C21	0.1 (3)
C2—C3—C4—C5	0.3 (4)	C16—C17—C22—C21	179.7 (2)
C3—C4—C5—C6	0.2 (3)	C18—C17—C22—C23	−179.2 (2)
C3—C4—C5—N1	178.6 (2)	C16—C17—C22—C23	0.5 (3)
C2—C1—C6—C5	1.1 (3)	C21—C22—C23—O2	−9.4 (3)
C2—C1—C6—C7	−178.1 (2)	C17—C22—C23—O2	169.8 (2)
C4—C5—C6—C1	−0.9 (3)	C21—C22—C23—C24	172.3 (2)
N1—C5—C6—C1	−179.54 (19)	C17—C22—C23—C24	−8.5 (3)
C4—C5—C6—C7	178.4 (2)	C16—C15—C24—C23	−4.1 (4)
N1—C5—C6—C7	−0.2 (2)	C14—C15—C24—C23	173.1 (2)
C1—C6—C7—N2	−59.5 (3)	C16—C15—C24—C7	−177.4 (2)
C5—C6—C7—N2	121.3 (2)	C14—C15—C24—C7	−0.3 (3)
C1—C6—C7—C24	58.2 (3)	O2—C23—C24—C15	−167.8 (2)
C5—C6—C7—C24	−121.0 (2)	C22—C23—C24—C15	10.5 (3)
C1—C6—C7—C8	177.7 (2)	O2—C23—C24—C7	4.4 (3)
C5—C6—C7—C8	−1.6 (2)	C22—C23—C24—C7	−177.30 (19)
N2—C7—C8—O1	57.6 (3)	N2—C7—C24—C15	−13.6 (2)
C6—C7—C8—O1	−179.4 (2)	C6—C7—C24—C15	−138.4 (2)
C24—C7—C8—O1	−55.0 (3)	C8—C7—C24—C15	107.1 (2)
N2—C7—C8—N1	−120.1 (2)	N2—C7—C24—C23	173.4 (2)
C6—C7—C8—N1	2.9 (2)	C6—C7—C24—C23	48.6 (3)
C24—C7—C8—N1	127.36 (19)	C8—C7—C24—C23	−65.9 (3)
N1—C9—C10—C11	72.8 (3)	O1—C8—N1—C5	179.1 (2)
C9—C10—C11—C12	76.8 (4)	C7—C8—N1—C5	−3.3 (2)
N2—C14—C15—C24	14.2 (3)	O1—C8—N1—C9	−4.3 (4)
N2—C14—C15—C16	−168.8 (2)	C7—C8—N1—C9	173.35 (19)
C24—C15—C16—O3	175.6 (2)	C4—C5—N1—C8	−176.2 (2)
C14—C15—C16—O3	−1.1 (4)	C6—C5—N1—C8	2.2 (2)
C24—C15—C16—C17	−4.2 (3)	C4—C5—N1—C9	7.1 (3)
C14—C15—C16—C17	179.0 (2)	C6—C5—N1—C9	−174.4 (2)
O3—C16—C17—C22	−173.9 (2)	C10—C9—N1—C8	−104.7 (3)
C15—C16—C17—C22	5.9 (3)	C10—C9—N1—C5	71.5 (3)
O3—C16—C17—C18	5.8 (4)	C15—C14—N2—C13	−156.7 (2)
C15—C16—C17—C18	−174.4 (2)	C15—C14—N2—C7	−23.2 (3)
C22—C17—C18—C19	−0.3 (4)	C6—C7—N2—C13	−77.4 (3)
C16—C17—C18—C19	−179.9 (2)	C24—C7—N2—C13	155.9 (2)
C17—C18—C19—C20	0.2 (4)	C8—C7—N2—C13	38.1 (3)
C18—C19—C20—C21	0.2 (4)	C6—C7—N2—C14	149.4 (2)
C19—C20—C21—C22	−0.4 (4)	C24—C7—N2—C14	22.8 (2)
C20—C21—C22—C17	0.3 (3)	C8—C7—N2—C14	−95.1 (2)

### Hydrogen-bond geometry (Å, º)

*Cg*1 is the centroid of the C1–C6 ring.

**Table:**

*D*—H···*A*	*D*—H	H···*A*	*D*···*A*	*D*—H···*A*
C1—H1···O1^i^	0.93	2.39	3.232 (4)	151
C9—H9*A*···O2^ii^	0.97	2.45	3.230 (3)	137
C20—H20···O1^iii^	0.93	2.52	3.205 (3)	131
C11—H11*B*···*Cg*1^iv^	0.97	2.82	3.759 (4)	164

Symmetry codes: (i) *x*, −*y*, *z*+1/2; (ii) −*x*+1/2, −*y*+1/2, −*z*+2; (iii) −*x*, *y*, −*z*+3/2; (iv) −*x*+1, *y*, −*z*+5/2.
